# Loss of Heterozygosity and Base Mutation Rates Vary Among *Saccharomyces cerevisiae* Hybrid Strains

**DOI:** 10.1534/g3.120.401551

**Published:** 2020-07-28

**Authors:** Ajith V. Pankajam, Suman Dash, Asma Saifudeen, Abhishek Dutta, Koodali T. Nishant

**Affiliations:** School of Biology, Indian Institute of Science Education and Research Thiruvananthapuram, India

**Keywords:** hybrid yeast, loss of heterozygosity, single nucleotide mutation, mitotic recombination, LOH hotspot

## Abstract

A growing body of evidence suggests that mutation rates exhibit intra-species specific variation. We estimated genome-wide loss of heterozygosity (LOH), gross chromosomal changes, and single nucleotide mutation rates to determine intra-species specific differences in hybrid and homozygous strains of *Saccharomyces cerevisiae*. The mutation accumulation lines of the *S. cerevisiae* hybrid backgrounds - S288c/YJM789 (S/Y) and S288c/RM11-1a (S/R) were analyzed along with the homozygous diploids RM11, S288c, and YJM145. LOH was extensive in both S/Y and S/R hybrid backgrounds. The S/Y background also showed longer LOH tracts, gross chromosomal changes, and aneuploidy. Short copy number aberrations were observed in the S/R background. LOH data from the S/Y and S/R hybrids were used to construct a LOH map for S288c to identify hotspots. Further, we observe up to a sixfold difference in single nucleotide mutation rates among the *S. cerevisiae* S/Y and S/R genetic backgrounds. Our results demonstrate LOH is common during mitotic divisions in *S. cerevisiae* hybrids and also highlight genome-wide differences in LOH patterns and rates of single nucleotide mutations between commonly used *S. cerevisiae* hybrid genetic backgrounds.

Mutations arise due to a variety of reasons ranging from errors in replication to the effect of DNA damaging agents. Mutations can include single base pair changes, small in-dels or large-scale deletions, loss of heterozygosity (LOH), gross chromosomal changes, and chromosomal aneuploidies. Mutations are among the primary sources of genetic variation but can also cause many genetic disorders (Phillips 2018; Svensson and Berger 2019). Since mutations are rare and subject to elimination by selection, it is often difficult to precisely estimate mutation rates. The classical method of measuring mutation rates is through a fluctuation assay using reporters (Luria and Delbruck 1943). The use of reporters to detect mutations has limitations (Baer *et al.* 2007). The method can be applied only to model organisms, and the measurement of mutation rates is restricted to a local region, which is then extrapolated genome-wide. In addition, the mutations are detected based on changes in a visible phenotype that is biased against mutations (*e.g.*, synonymous or in non-coding regions) that do not result in phenotypic differences. Another standard method involves the use of mutation accumulation lines (MA lines). Multiple independent lines are propagated from a common ancestor for a large number of generations to facilitate the accumulation of rare mutational events. The effect of natural selection is minimized (via bottlenecks) so that nearly all non-lethal mutations can be recovered. The sequences of the ancestor and the MA lines can be compared with high throughput sequencing to obtain precise and unbiased estimates of genome-wide mutation rates (Keightley and Halligan 2009).

MA experiments have been previously used in *S. cerevisiae*, *Drosophila*, *C. elegans*, and other organisms to estimate spontaneous mutation rates (Denver *et al.* 2000; Denver *et al.* 2004; Lynch *et al.* 2008; Keightley *et al.* 2009; Nishant *et al.* 2010; Burke *et al.* 2010; Ness *et al.* 2015; Dutta *et al.* 2017; Flynn *et al.* 2017). *S. cerevisiae* is particularly suited for mutation accumulation study as it is a eukaryote with short generation time (∼2.5 hr for mitosis), small genome size (12 Mb), and can be propagated in either haploid or diploid state. Most mutation rate estimates in *S. cerevisiae* have come from homozygous backgrounds (Wloch *et al.* 2001; Joseph and Hall 2004; Lynch *et al.* 2008; Nishant *et al.* 2010; Zhu *et al.* 2014; Liu and Zhang 2019). But homozygous backgrounds also mask major genomic changes like LOH that contribute to the mutational process (Dutta *et al.* 2017; Flynn *et al.* 2017). Moreover, recent studies suggest that about 63% of natural *S. cerevisiae* diploid isolates are heterozygous, which suggests LOH can play a major role in genetic variation in *S. cerevisiae*. (Peter *et al.* 2018; James *et al.* 2019). LOH is one of the most potent modes of genome evolution and can contribute significantly more to genetic variation than base mutations (Gerstein *et al.* 2014; Ene *et al.* 2018; James *et al.* 2019). Further, there is evidence that suggests heterozygous genomes may be more mutagenic (Amos 2010; Yang *et al.* 2015). Therefore, the mutation rate estimates in *S. cerevisiae* from homozygous backgrounds may not be applicable in a heterozygous context. To understand mutation rates in the context of heterozygous genomes, we previously analyzed *de novo* single nucleotide mutations (SNMs) and LOH in a heterozygous *S. cerevisiae* genome generated by crossing two strains S288c and YJM789 (Dutta *et al.* 2017). Although average SNM rates in the S288c/YJM789 hybrid (1.82 × 10^−10^ per base per cell division), were similar to homozygous *S. cerevisiae* S288c strain (1.67 × 10^−10^ per base per cell division), surprisingly we observed more LOH tracts than expected in two of the five S288c/YJM789 hybrid lines, given known mitotic recombination rates in *S. cerevisiae* (Dutta *et al.* 2017). These results showed that mitotic recombination in heterozygous genomes can cause significant genotypic changes over a large number of generations through LOH.

Recent studies have shown mutation rate variation among *S. cerevisiae* strains using a single reporter based fluctuation assay (Gou *et al.* 2019). In addition, recent studies have also analyzed LOH and *de novo* mutations genome-wide in *S. cerevisiae* heterozygous strains undergoing experimental evolution (James *et al.* 2019). Analysis of *S. cerevisiae* and *S. paradoxus* intra and interspecies hybrids have shown LOH rates are higher for intraspecies hybrids (with lower heterozygosity) relative to interspecies hybrids while the opposite is true for SNM rates (Tattini *et al.* 2019).

To understand differences in LOH and SNM rates between *S. cerevisiae* strains, we performed a genome-wide analysis of MA lines using hybrid and homozygous strains of *S. cerevisiae*. Our experimental setup facilitated a comparison of LOH rates and patterns among *S. cerevisiae* hybrids genome-wide. It also facilitated the comparison of single nucleotide mutations and gross chromosomal changes in both heterozygous and homozygous context in *S. cerevisiae*. We set up MA lines with two different hybrids S288c/YJM789 (S/Y) and S288c / RM11-1a (S/R) with similar heterozygosity and the homozygous diploids S288c (SS), RM11 (RR), and YJM145 (YY) respectively (Mortimer and Johnston 1986; Tawfik *et al.* 1989; Mortimer *et al.* 1994). *S. cerevisiae* hybrids S/Y and S/R are widely used for genome-wide meiotic recombination analysis in the laboratory because these strains have a high density of heterozygous SNPs (∼50,000 SNPs in 12 Mb genome) uniformly distributed in the genome that facilitates analysis of recombination events (Mancera *et al.* 2008; Qi *et al.* 2009; Anderson *et al.* 2011; Oke *et al.* 2014; Krishnaprasad *et al.* 2015; Anderson *et al.* 2015; Chakraborty *et al.* 2017). All MA lines were propagated for 100 bottlenecks. We observed extensive LOH in both S/Y and S/R backgrounds and used the data to construct a LOH map for the S288c genome. Further, LOH patterns and single nucleotide mutation (SNM) rates were different among the two hybrid backgrounds. Our results show that different hybrid backgrounds of *S. cerevisiae* can show considerable variation in SNM rates and LOH signatures depending on their genetic background.

## Materials And Methods

### Media and strains

The MA lines were grown on YPD (yeast extract, peptone, and dextrose) media at 30° (Rose *et al.* 1990). The two hybrid combinations (S/R and S/Y) were made by mating the corresponding haploids of the opposite mating type. Diploids were selected using the auxotrophic markers and by testing for sporulation. The genotypes of the diploid *S. cerevisiae* strains used for this study are: S/Y hybrid (KTY83 x KTY84) (*MATα **ho*::*hisG **lys2** cyh* / *MATa **ho* *lys5*); S/R hybrid (KTY188 x KTY277) (*MATα **his3**Δ **leu2**Δ **lys2**Δ **ura3**Δ / MATa **leu2**Δ0 **ura3**Δ0 **HO*::*KanMX*); RR (YJM1293); YY (YJM145); SS (KTY186 x KTY188) (*MATa **his3**Δ **leu2**Δ **met15**Δ **ura3**Δ / MATα **his3**Δ **leu2**Δ **lys2**Δ **ura3**Δ*).

### Maintaining MA lines

From single colonies of each hybrid parent (S/R and S/Y), 10 replicate lines were initiated. Five replicate lines were initiated from each of the homozygous diploid parents (SS, YY, RR). Each MA line was propagated by streaking a single colony onto a new plate of YPD agar every two days. Colony selection was randomized. In the 20^th^, 40^th^, 60^th^, 80^th^ and 100^th^ bottlenecks, samples from overnight YPD patches of each transferred colony were frozen at -80° in 20% glycerol. The sequence analysis was performed only on the samples frozen after 100 bottlenecks and the parent strains.

### Spore viability and sporulation efficiency

Sporulation efficiency of the parent strains was determined by monitoring Meiosis I (MI) and Meiosis II (MII) divisions, as described in Chakraborty *et al.* 2017. Culture samples were withdrawn at periodic intervals until 24 hr, and the cells visualized under the fluorescence microscope after DAPI staining. The sporulation efficiency was calculated as the fraction of cells in MI (2-3 nuclei stage) and MII (4 nuclei stage) to the total number of cells counted (∼200). For analysis of spore viability, the diploids were patched on sporulation media. After two days of incubation at 30°, tetrads were dissected on synthetic complete medium using a Zeiss dissection microscope.

### DNA extraction and sequencing

The parent strains and colonies from their respective MA lines (100^th^ bottleneck only) were independently cultured overnight at 30° in YPD liquid medium. The DNA was isolated from each culture using the PrepEase DNA isolation kit from Affymetrix following the manufacturer’s protocol. The genomic DNA was sequenced using Illumina Hi- Seq 2500 platform at Fasteris, Switzerland.

### Read mapping, genotyping of whole-genome sequencing data

The sequence reads from the 35 MA lines, and the parent strains were mapped to the S288c genome (version 64-1-1, 2011) using bowtie2 (version 2.1.0) (Langmead and Salzberg 2012). Uniquely mapped reads were considered for the SNP calling (duplicate reads were removed using picardtools). To reduce misalignment due to indels, we performed a local indel realignment after mapping the reads to the reference genome using GATK IndelRealigner. SNPs from all genomic regions were called in the S/Y and S/R hybrid lines using GATK unified genotype caller. R packages were used for data visualization.

### Analysis of LOH and gross chromosomal changes

For analysis of LOH, heterozygous SNP positions were determined in the S/Y and S/R parent genomes. Fixed SNPs (not shared as homozygous in >50% of the MA lines) were identified at these positions using custom R scripts.

LOH tracts were called in all the S/Y and S/R MA lines if supported by two or more fixed SNPs. LOH tracts supported by 10 or more fixed SNPs were also separately called (Dutta *et al.* 2017).

The tract size was determined by taking the distance between the mid-points of the 1^st^ het SNP flanking each LOH border. To determine LOH hotspots, we partitioned the genome into 1 bp bins and plotted the count of the LOH tracts (S288c tracts) that are overlapping with these bins.

To analyze gross chromosomal changes, the genome was partitioned into 5 kb bins, and the read counts in each bin were plotted using custom R scripts. The median and 2x median read coverage were also plotted.

### Analysis of single nucleotide mutations

Whole-genome sequences of the 35 MA lines was compared with the parent sequences to detect new SNMs. Reads having mapping quality < 40 were removed prior to analysis. Mutations were called using muver (Burkholder *et al.* 2018), accuMUlate (Winter *et al.* 2018), and methods described in Dutta *et al.* 2017. Only mutations that were commonly called from these methods were considered. We further filtered away mutations that are not supported by at least 40 reads. Out of 122 new mutations, 20 mutations representing the five lines were verified by Sanger sequencing.

### Data availability

All parent strains and MA lines are available upon request. Sequence data for the 35 MA lines and the parent strains (S/R_P, YY_P, SS_P, RR_P) are deposited in the National Centre for Biotechnology Information Sequence Read Archive under accession number: SRP254814 (Bioproject accession number: PRJNA622400). For S/Y parent (S/Y_P), sequence data are available under accession number SRP098673. Supplementary figures include Figure S1: Analysis of LOH (supported by 10 or more SNPs) in S/Y and S/R hybrids; Figure S2: Frequency of heterozygous SNPs in S/Y and S/R hybrids; Figure S3: Analysis of read counts mapping to the chromosomes in the MA lines and parent strains; Figure S4: Verification of SNMs by Sanger sequencing; Figure S5: Sporulation kinetics of S/Y, S/R, RR, YY, SS parent strains and S/Y_8 line. Supplementary tables include Table S1: Summary of sequencing statistics for all 35 MA lines and the parent strains; Table S2: SNP counts in vegetative lines of S/Y hybrids; Table S3: SNP counts in vegetative lines of S/R hybrids; Table S4: Distribution of 316 LOH tracts (supported by two or more SNPs) in the S/Y hybrid; Table S5: Distribution of 942 LOH tracts (supported by two or more SNPs) in the S/R hybrid; Table S6: Distribution of 101 LOH tracts (supported by 10 or more SNPs) in the S/Y hybrid; Table S7: Distribution of 50 LOH tracts (supported by 10 or more SNPs) in the S/R hybrid; Table S8: Spore viability and aneuploidy data of the 35 MA lines and their parent strains; Table S9: Single nucleotide mutations in the 35 MA lines. Supplemental material available at figshare: https://doi.org/10.25387/g3.12643310.

## Results

### Mutation accumulation lines of S. cerevisiae hybrid and homozygous strains

**Figure 1:**
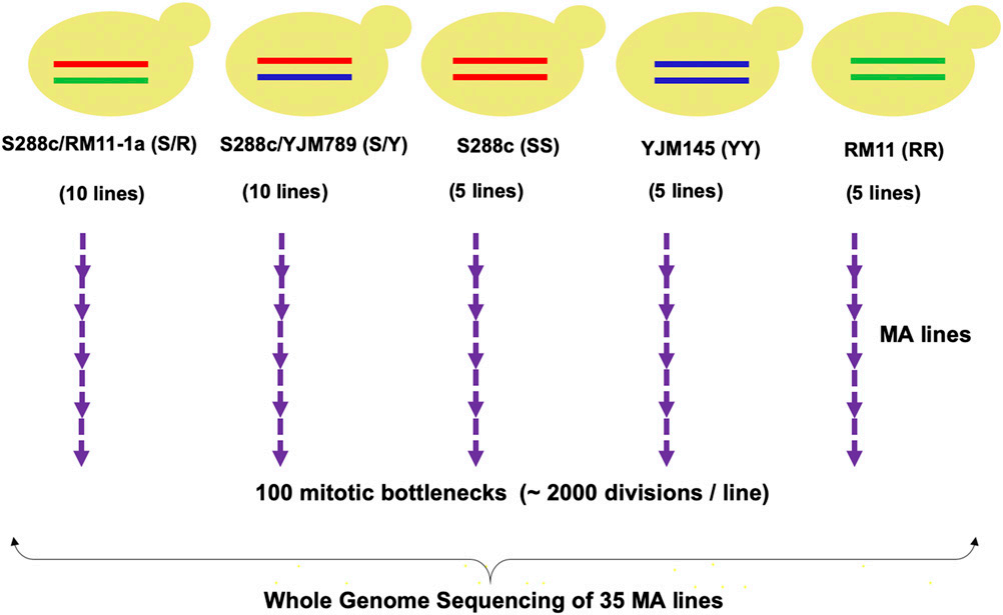
Experimental set up of the mutation accumulation lines. 10 lines each of the *S. cerevisiae* S/R, S/Y hybrids and five lines each of the diploid homozygous *S. cerevisiae* strains, SS, YY and RR were propagated for 100 bottlenecks. Whole-genome sequences of the 35 MA lines after 100 bottlenecks were compared to the respective parent genomes to detect single nucleotide mutations, LOH, aneuploidy, and other gross chromosomal changes.

Vegetative mutation accumulation lines of the S/Y hybrid, S/R hybrid, and the diploid homozygous strains, SS, YY, and RR were set up as described in Materials and Methods (Figure 1 and (Nishant *et al.* 2010)). For S/Y and S/R hybrids, 10 lines were bottlenecked to single cells from a colony every two days (20 generations) and propagated for a total of 100 bottlenecks (∼2000 generations). These lines were labeled S/Y_1 to S/Y_10 (S/R_1 to S/R_10). For SS, YY, and RR homozygous diploids, five lines each were propagated for the same number of bottlenecks (100 bottlenecks, ∼2000 generations) as the hybrid lines. These lines were labeled SS_1 to SS_5, YY_1 to YY_5, and RR_1 to RR_5. All the MA lines were stocked every 20 bottlenecks. Whole-genome sequencing of all the 35 MA lines was performed after 100 bottlenecks. The parent strains for S/R, SS, YY, and RR lines were also sequenced (S/Y parent sequence was from Dutta *et al.* 2017). Sequencing details for all 40 genomes are in Table S1. The average sequencing depth was ∼70X (Table S1).

### LOH patterns are distinct between the S. cerevisiae hybrids

**Figure 2:**
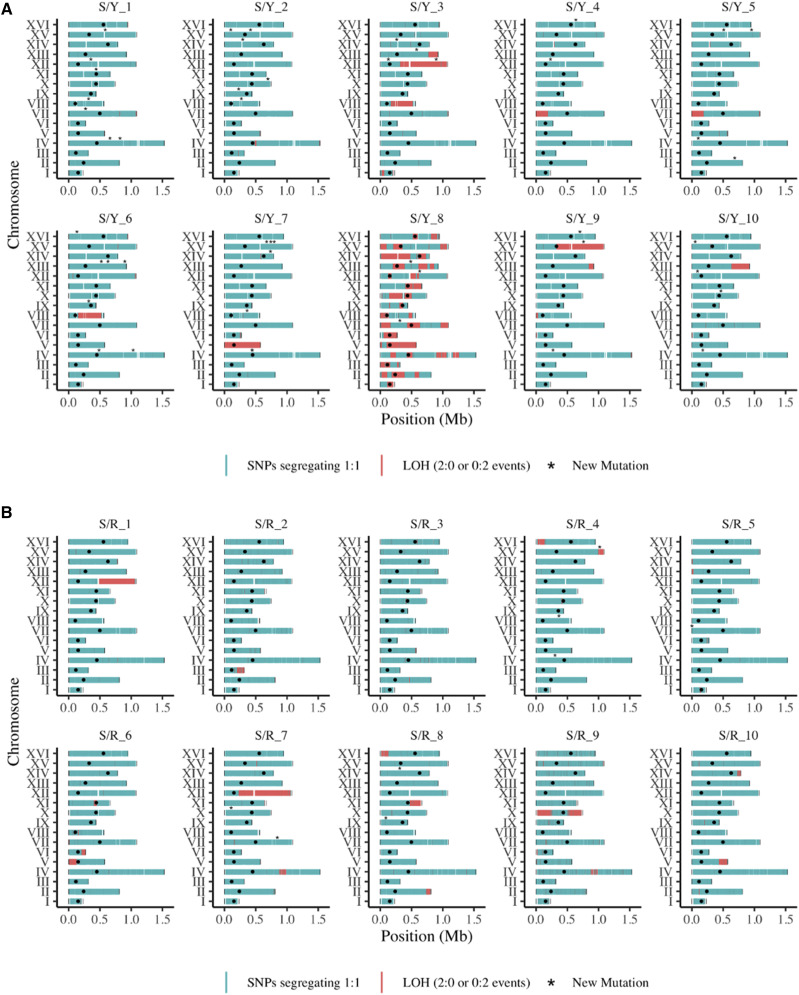
Genome-wide LOH plots for the *S. cerevisiae* S/Y and S/R hybrids. A) LOH plot for S/Y lines, B) LOH plot for S/R lines. Regions with LOH are in orange (2:0 or 0:2), and heterozygous regions are in cyan (1:1). Centromeres are shown in black dots, and SNMs are shown in asterisk (*).

Previous studies from our laboratory analyzed LOH in five vegetative lines of the S/Y hybrid maintained for 57 bottlenecks by following a set of 47954 SNPs common in all five lines (Dutta *et al.* 2017). The frequency of LOH tracts in three of these lines was consistent with known rates of mitotic recombination (Lee *et al.* 2009; Rosen *et al.* 2013; St Charles and Petes 2013; Yin and Petes 2013; Yim *et al.* 2014), but two of the lines showed extensive LOH involving 22–35% of the SNPs (Dutta *et al.* 2017). To more extensively characterize LOH in the S/Y hybrid and to compare LOH rates and patterns in S/Y with another hybrid, we analyzed LOH data in 10 S/Y and 10 S/R hybrids that were maintained for 100 bottlenecks. The two hybrids share the S288c background, which shows 0.5–0.6% divergence with the YJM789 and RM11 strain background. SNP data from the diploid S/Y and S/R lines were used to call LOH genome-wide (Table S2 and Table S3). We genotyped 51,399 SNPs common in all S/Y hybrid lines and 41,162 SNPs common in all S/R hybrid lines. Recent studies have shown that LOH involving single nucleotides (SLOH) represents a significant class of LOH events in organisms like *Candida albicans* and *Daphnia pulex* (Keith *et al.* 2016; Ene *et al.* 2018). We called LOH tracts if they were supported by two or more SNPs in S/Y and S/R hybrids because of the possibility that some of the single SNP based LOH may be artifacts (Flynn *et al.* 2017). We observed 316 LOH tracts in S/Y, and 942 LOH tracts in S/R, suggesting LOH contributes significantly to genetic variation (Table S4, S5). Genome-wide plot of the distribution of LOH tracts in the twenty S/Y and S/R hybrids is shown in Figure 2. S/Y_8 showed significantly more LOH tracts compared to other S/Y lines suggesting it could be an outlier (Figure 2). Among the 316 LOH tracts in S/Y hybrids, 98 LOH tracts came from S/Y_8 (Table S4). Such outliers with a high frequency of LOH in S/Y hybrids have been observed previously also (Dutta *et al.* 2017). Combining LOH data from the 10 S/Y lines in this study with the previously analyzed five S/Y lines (Dutta *et al.* 2017), three out of 15 S/Y lines (20%) show significantly more LOH than other S/Y lines. Among the S/R hybrids, three outliers with enhanced LOH were observed (Table S5). These include S/R_2 (138 LOH tracts), S/R_3 (151 LOH tracts), and S/R_9 (342 LOH tracts). LOH tracts from these S/R outliers and other S/R lines are not apparent in the genome-wide plot as the tract sizes are smaller in the S/R hybrid (Figure 2, see below).

We also called LOH tracts conservatively if they were supported by a minimum of 10 SNPs (Dutta *et al.* 2017). This method will however, exclude very short LOH tracts. We observed 101 LOH tracts in the S/Y lines after 100 bottlenecks (Table S6). About half of these tracts came from S/Y_8 (54 LOH tracts), which again shows that it has undergone significantly more LOH than other S/Y lines. Fewer LOH tracts (50) were observed in the S/R hybrid (Table S7), suggesting that most tracts in S/R hybrid were short and not detected with a SNP cutoff of >10.

**Figure 3:**
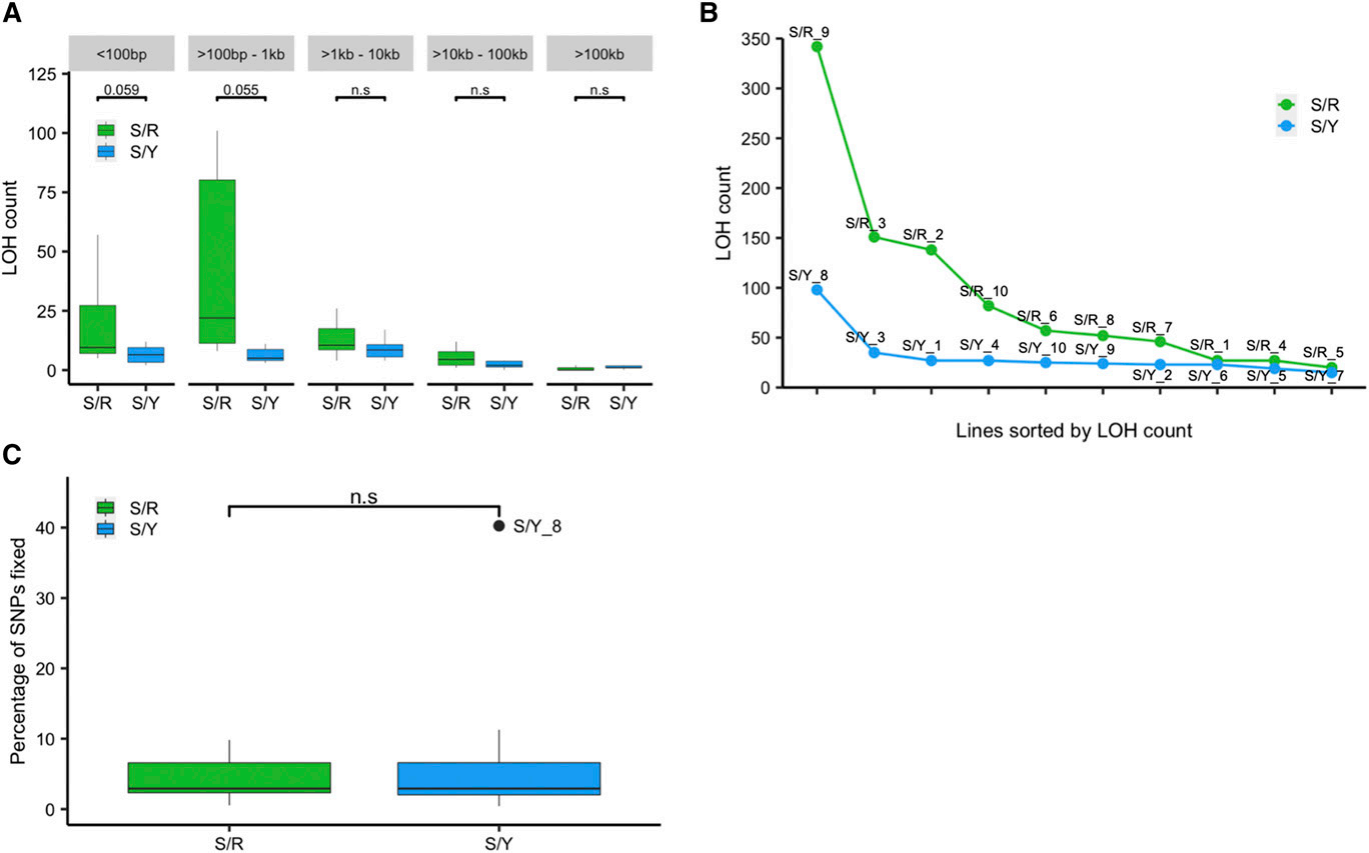
Analysis of LOH in S/Y and S/R hybrids. A) Box plot showing the number of LOH tracts (supported by two or more SNPs) among different classes of LOH tract sizes in S/Y and S/R hybrids. Outliers are removed. Non-significant differences are shown as n.s (*t*-test). B) Individual S/Y and S/R lines sorted based on the number of LOH counts (supported by two or more SNPs). C) Percentage of SNPs fixed relative to the number of heterozygous SNP positions in the S/R and S/Y hybrids. n.s indicates non-significant (*t*-test).

We compared LOH tract sizes between the S/Y and S/R lines using a range from less than 100 bp to over 100 kb (Figure 3A). We observe the smaller LOH tract sizes (< 1 kb) are common in S/R lines while larger LOH tract sizes (>1 kb) are common in S/Y lines (Figure 3A, Figure S1A). LOH count data (tracts supported by two or more SNPs) for individual lines show an enhanced number of LOH in the S/R lines with S/R_2, S/R_3, and S/R_9 as outliers (Figure 3B). Fewer LOH tracts were observed in the S/Y lines with S/Y_8 as an outlier (Figure 3B). For LOH tracts supported by 10 or more SNPs similar numbers of LOH tracts are observed for S/R and S/Y with S/Y_8 as an outlier (Figure S1B). The median LOH tract length for S/Y was 1.73 kb (average: 28.40 kb), and for S/R the median LOH tract length was 247 bp (average: 4.03 kb). These results also suggest LOH tracts are significantly longer in S/Y relative to the S/R hybrid (*P* <0.001, Wilcoxon rank-sum test). A heat map showing the frequency of heterozygous SNPs also showed longer LOH tracts in S/Y compared to S/R hybrids (Figure S2). We separately analyzed interstitial LOH tracts (not extending to within 100 bp of the telomere), since terminal tracts are likely crossovers that can span much longer distances from a single mitotic DSB repair event. These interstitial tracts most likely reflect DSB repair by gene conversions. The median tract length for interstitial LOH events in S/Y (1.7 kb) was also significantly greater than S/R (242.25 bp) (< 0.001, Wilcoxon rank-sum test). These differences in LOH tract lengths between the S/Y and S/R hybrids are unlikely to result from differences in marker density since the median distance for markers in S/Y and S/R hybrids is similar (93 bp and 102 bp respectively). For the LOH tracts supported by a minimum of 10 SNPs, we observed contiguous LOH tracts where switching was observed between the S288c and YJM789 haplotypes in three S/Y lines (S/Y_8, S/Y_9, and S/Y_10) (Table S6). In the S/R lines, we observed contiguous LOH tracts that showed switching between the S288c and RM11 haplotypes in S/R_8 (Table S7). Since the LOH tracts were analyzed after 100 bottlenecks, it is uncertain whether the haplotype switching resulted from the same DSB repair event or from distinct DSB repair events during the mitotic divisions.

We observed a single instance in S/Y_7 line where the entire chromosome V had undergone LOH (Figure 2A). This may be a consequence of recombination independent mechanisms like chromosome loss followed by duplication. It has been hypothesized that when diverged yeast species hybridize in the wild, recombination independent LOH mechanisms drive genome homogenization (Morales and Dujon 2012). On average, LOH tracts in S/Y hybrids were closer to the telomere (139.86 kb) than the centromere (258.13 kb) (Table S4). This observation is consistent with previous findings on LOH tracts in the S/Y hybrid (Dutta *et al.* 2017). It is also consistent with previous observations that mitotic recombination rates are higher in centromere distal regions (Mandegar and Otto 2007; Andersen *et al.* 2008). LOH tracts in S/R hybrids were also observed closer to telomeres (179.31 kb) than centromeres (270.81 kb) (Table S5). Since all terminal (crossover) LOH tracts span the telomeres even if the recombination event happened far from the telomere, we also examined the distribution of interstitial LOH tracts. These were also observed to be closer to the telomeres (84.07 kb) than centromeres (182. 99 kb) in S/Y and in S/R (204.89 kb from centromeres, 142.93 kb from telomeres) hybrids. However, in some S/Y (*e.g.*, S/Y_8) and S/R lines, the LOH tracts were observed to span the centromere suggesting centromeres are not immune to LOH mediated by mitotic recombination (Figure 2A, Table S4, Table S5) (Zafar *et al.* 2017).

We also compared the fixation of total SNPs between S/Y and S/R independent of tract lengths. A similar portion of the heterozygous SNPs was observed to be fixed in both S/Y (mean 7.51%, median: 2.91%) and S/R (mean 4.33%, median 2.91%) backgrounds with no statistically significant difference (Figure 3C). Among all 20 S/Y and S/R lines, the S/Y_8 line showed a significantly higher percentage of fixed SNPs (40.26%) (Figure 3C). To account for differences in SNP density between the two hybrids, we also estimated the percentage genome fixed. The percentage genome fixed for the S/Y hybrid (median 3%, mean 7.4%) and the S/R hybrid (median 2.17%, mean 3.14%) were not statistically different (Binomial test). Overall, these results suggest significant LOH in both S/Y and S/R backgrounds with a few outliers in each background. Further, there are differences in LOH patterns between S/Y and S/R hybrids. The S/Y hybrids show longer tracts suggesting different mechanisms may cause variations in the LOH spectrum across different genetic backgrounds (see discussion).

**Figure 4:**
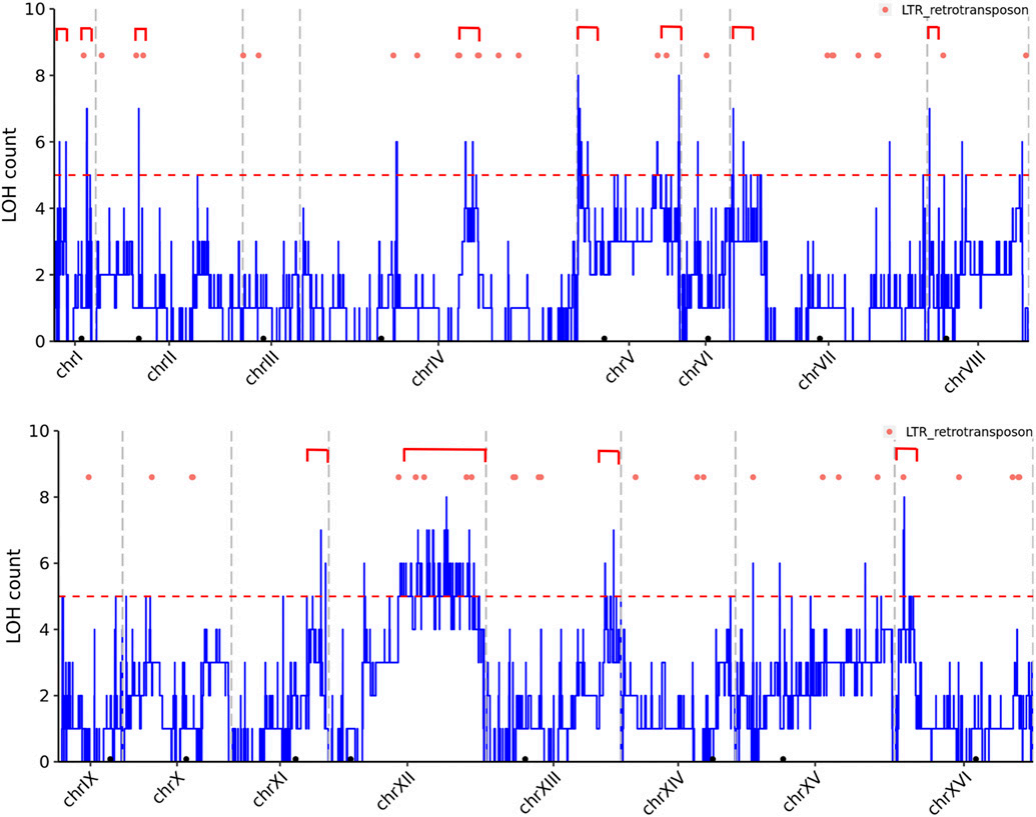
Chromosomal map of LOH for *S. cerevisiae* S288c strain. Total count of LOH tracts from 15 S/Y (includes data from 5 S/Y hybrids from Dutta *et al.* 2017) and 10 S/R hybrids with their positions on each chromosome. Centromeres are shown in black dots. Chromosome boundaries are shown in gray vertical dash lines. Dash red lines show LOH count of 5 (shared in 20% of the lines). LOH hotspots are shown in red rectangle brackets. Orange dots show LTR retrotransposon positions.

We used the LOH data from the 20 MA lines of S/Y and S/R hybrids and the five previously sequenced S/Y lines (Dutta *et al.* 2017) representing a total of 1399 LOH events to construct a LOH map for *S. cerevisiae* S288c strain (Figure 4). The LOH map validated chromosomal regions with enhanced LOH observed in previous studies -e.g chromosome IV right arm and the rDNA cluster on the right arm of Chromosome XII (Smukowski Heil *et al.* 2017; Fisher *et al.* 2018; James *et al.* 2019). Further, we observe additional LOH hotspots from our map. These include the left arm of chromosome VII and XVI and the right arm of chromosomes XI and XIII toward the telomeric end; the telomeric ends on both left and right arms of chromosome I and V; near the centromere on chromosome II. In general, for most chromosomes, LOH rates are elevated toward the chromosomal ends. Some of the new LOH hotspots on chromosomes I, II, and XVI are in the vicinity of LTR retrotransposon repeats (Ty elements) (Figure 4). LOH counts are also observed to be reduced for specific chromosomes like chromosome III (Figure 4).

### Gross chromosomal changes and aneuploidies

**Figure 5:**
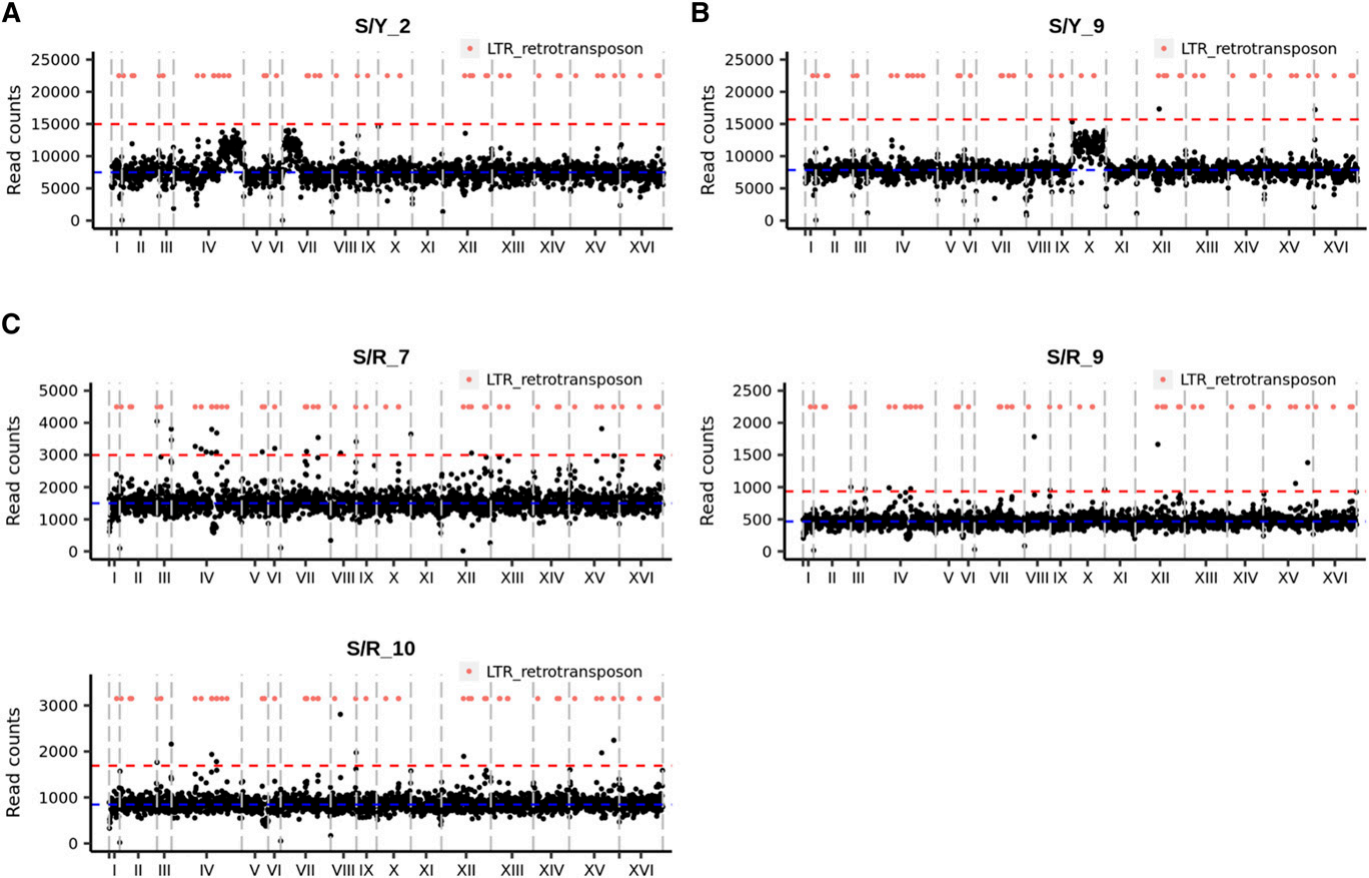
Analysis of gross chromosomal changes in S/Y and S/R hybrids. A) Segmental duplications (Chromosomes IV and VII) in S/Y_2. B) Chromosome X aneuploidy in S/Y_9. C) Segmental deletions in S/R_7, S/R_9, and S/R_10. Gray vertical lines show chromosomal boundaries. Horizontal blue and red lines show the median and 2x median read counts that map to the chromosomes respectively. Orange dots show LTR retrotransposon positions in all panels.

In addition to LOH, we also analyzed gross chromosomal changes involving segmental duplication and deletions (copy number aberrations) in the S/Y and S/R hybrids. Segmental duplications involving chromosome IV and VII were observed in the S/Y_2 line (Figure 5A, Figure S3). These were seen as half aneuploidies on coverage plots for S/Y_2. Whole chromosomal aneuploidy involving gain of specific chromosome (chromosome X trisomy) was also observed in the S/Y_9 line (Figure 5B, Figure S3, Table S8). We also found evidence for segmental deletions on chromosome IV in S/R_7 and S/R_9 and on chromosome V in S/R_10 (Figure 5C). These segmental deletions were also flanked by LTR retrotransposon repeats (Ty elements). Along with the LOH data, these results reveal the S/Y and S/R hybrid genomes are dynamic during mitotic divisions.

Aneuploidy is common in *S. cerevisiae*, and in laboratory conditions, the spontaneous rate of individual chromosome loss ranges from 10^−6^ to 10^−7^ / cell division (Kumaran *et al.* 2013; Mulla *et al.* 2014; Peter *et al.* 2018; Gilchrist and Stelkens 2019). Among the homozygous lines, aneuploidy was observed in the RR, YY lines, but not in the SS line (Figure S3, Table S8). All five RR lines showed aneuploidy involving chromosome IX. Coverage plots for the RR parent showed slightly enhanced coverage for chromosome IX, suggesting a subset of the cells may be aneuploid in the diploid parent. This will likely explain the aneuploidy of chromosome IX in all the five RR lines. Two RR lines also showed an additional aneuploidy involving chromosome XI (Figure S3, Table S8). Aneuploidy involving chromosome XII was observed in one of the five YY lines. Although both the RR and YY lines showed aneuploidy, no significant differences were observed in their viability compared to the parent strains after 100 bottlenecks (Table S8). These results suggest that extra copies of these chromosomes (IX, XI, XII) do not confer viability defects. Further, chromosomes IX and XI are also observed to be frequently aneuploid (Gilchrist and Stelkens 2019). These results are consistent with the observation that many wild strains show aneuploidy, but without growth defects (Peter *et al.* 2018; Hose *et al.* 2020). Since aneuploidy was not observed in the S/R hybrid or the SS line, it is possible that factors contributing to the enhanced aneuploidy in RR are suppressed in the presence of the S288c genome. Similarly, the chromosomal aneuploidy observed in S/Y hybrid may be due to the YJM789 genome.

### Genome-wide SNM rates are different in the S. cerevisiae hybrid backgrounds

**Figure 6:**
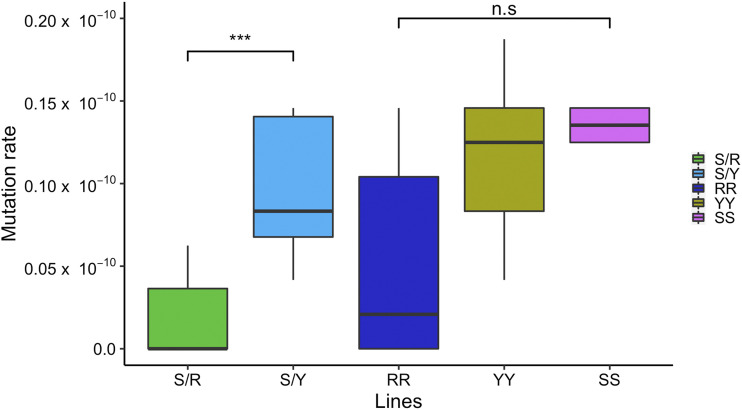
SNM rate in *S. cerevisiae* MA lines. The box plot shows the SNM rate in the two hybrids (S/Y, S/R) and the diploid homozygous lines (RR, YY, SS). “***” indicates p-value <0.001 (*t*-test). n.s indicates non-significant differences.

We estimated differences in the number of SNMs among all five genetic backgrounds comprising the two hybrid and three homozygous lines (S/Y, S/R, RR, SS, YY). SNMs were detected in each of the lines after 100 bottlenecks with reference to their respective parent diploid genome. We observed 8 SNMs in S/R and 47 in S/Y. Among the isogenic lines, we observed 13 SNMs in RR; 26 in SS; 28 in YY. SNMs were called conservatively (materials and methods), and out of 122 new mutations, all 20 mutations tested by Sanger sequencing were validated (Figure S4). All the SNMs in the 35 MA lines were heterozygous, and many were in coding sequences (Table S9, see discussion). The 35 MA lines were propagated for a total of ∼2000 mitotic divisions (100 bottlenecks), and the 24.04 Mb genome was sequenced at 99.9% sequence coverage. The average SNM rates for each of the genetic backgrounds are (S/R: 0.17 × 10^−10^; S/Y: 0.98 × 10^−10^; RR: 0.54 × 10^−10^; SS: 1.35 × 10^−10^; YY: 1.16 × 10^−10^) (Figure 6, Table S9). The number of mutations and mutation rates for each of the lines is shown in Table S9. The average SNM rate for S/Y (0.98 × 10^−10^) is comparable to the earlier estimate in the S/Y hybrid (1.82 × 10^−10^, Dutta *et al.* 2017). The average SNM rate in the SS homozygous diploid (1.38 × 10^−10^) is also similar to SNM rate estimates (1.67 × 10^−10^ per base per generation) from a large set of 145 diploid *S. cerevisiae* vegetative mutation accumulation lines (Zhu *et al.* 2014).

A comparison of the SNM rates between the five genetic backgrounds shows heterogeneity. Between the two hybrids (S/R and S/Y), the S/Y hybrid showed approximately sixfold (5.76) higher SNM rate (*P* < 0.001, *t*-test) (Figure 6). Overall a maximum of an eightfold difference exists between SNM rates in S/R compared to the rate in SS. These results show that differences in the genetic background affect the genome-wide SNM rate in the *S. cerevisiae* strains. Among the homozygous lines, there were no statistically significant differences in SNM rates between RR, SS, and YY. Recent studies using single-locus fluctuation assays have shown that the haploid RM11 (RM11-1a) strain is more mutagenic compared to S288c (Gou *et al.* 2019). This effect is mediated by polymorphisms in the *RAD5* gene, which is involved in post replication DNA repair (Demogines *et al.* 2008). *rad5* mutants show enhanced sensitivity to DNA damaging agents and enhanced recombination (Friedl *et al.* 2001). In our study, SNM rates in the diploid RM11 strain were not significantly different from S288c, although enhanced aneuploidy was observed in the diploid RM11. These results are consistent with differences in mutation rates and spectrum in haploid and diploid backgrounds for the same strain with more SNMs in haploids and larger structural changes in diploids (Sharp *et al.* 2018).

## Discussion

Meiosis is infrequent in *S. cerevisiae* and usually involves intra-tetrad mating *(*Tsai *et al.* 2008). Therefore, mitotic recombination and LOH constitute a major mechanism for maintaining genetic variation in *S. cerevisiae* (Peter *et al.* 2018). Further, in asexual organisms, LOH through mitotic recombination plays a significant role in genome evolution (Dale *et al.* 2019). LOH events occur 1000 times more frequently than SNMs (Omilian *et al.* 2006). LOH is considered to be a stress response facilitating rapid adaptation as it allows for non-dominant and rare variants to express and therefore is also termed as fitness associated recombination (Forche *et al.* 2011; Gerstein *et al.* 2014; Peter *et al.* 2018). However, most MA experiments in *S. cerevisiae* have used homozygous lines missing this important source of genetic variation. Further, most estimates of LOH rates in *S. cerevisiae* and other yeasts have come in the context of experimental evolution studies, which could affect both the occurrence as well as detection of all LOH events due to selection. There is, therefore, a strong need for more MA experiments in heterozygous *S. cerevisiae* models to quantify LOH rates and patterns.

Previous studies in *S. cerevisiae* have demonstrated LOH events outnumber SNMs, but in an experimental evolution context, with selection favoring LOH over SNMs (James *et al.* 2019). Cumulative data from our earlier work (Dutta *et al.* 2017) and the present study suggests extensive LOH in both S/Y and S/R hybrid backgrounds and show that LOH (1258 events) can contribute significantly more to genetic change than SNMs (122 events) even with minimal selection. In the present study, we analyzed LOH in two different *S. cerevisiae* hybrid combinations involving S228c (S/Y and S/R) that have similar heterozygosity. Our results suggest that the extent of LOH is similar between the two hybrids (Figure 3C) and independent of the genetic background (James *et al.* 2019). Although LOH was extensive in both S/Y and S/R backgrounds, we observed differences in the pattern of LOH tracts. S/Y hybrids showed longer LOH tracts than S/R hybrids. LOH tract lengths have been shown to vary depending on the nature of stress in *Candida albicans* (Forche *et al.* 2011). Further, *S. cerevisiae* clinical isolates are also known to have long LOH tracts (Magwene *et al.* 2011). It may also be that one hybrid has longer DSB resection, or maybe one hybrid is able to do longer tracts of 3′ extension (BIR-like). These differences may also result in different tract lengths. It is, therefore, likely that differences in the genetic background of S/Y and S/R result in different LOH tract lengths. Out of 51,399 and 41,162 SNP positions called out in the S/Y and S/R hybrids, only 24,653 SNP positions are common between the two hybrids, while the rest are unique suggesting S/Y and S/R represent distinct genetic backgrounds.

We also observed outliers in both S/Y and S/R backgrounds that had significantly more LOH than the other lines. One possible explanation for the LOH pattern (with long internal tracts) in outliers like the S/Y_8 line (40.26% fixed SNPs) is the occurrence of RTG (return to growth) events where cells transiently enter meiosis and undergo programmed DSBs, but complete the repair of the DSBs during return to mitotic growth (Dayani *et al.* 2011; Laureau *et al.* 2016; Dutta *et al.* 2017; Brion *et al.* 2017). We compared the sporulation efficiencies of the S/Y_8 line with the parent strains (S/Y, S/R, RR, SS, and YY) in meiotic time courses (Figure S5). The RR diploid showed good sporulation (69% after 24 hr), while the SS and YY diploid showed poor sporulation (4% and 21% respectively after 24 hr). The hybrids S/Y and S/R displayed intermediate sporulation efficiencies (36% and 67% respectively after 24 hr). These results suggest the S/R hybrid is a better sporulator than S/Y. The S/Y hybrid showed improved sporulation relative to the parent strains suggesting heterosis in sporulation efficiency. The sporulation efficiency of the S/Y_8 line (16%) was less compared to the S/Y and S/R parents after 100 bottlenecks. It is likely that enhanced LOH in S/Y_8 and other outliers reflects extensive mitotic recombination in a subset of the lines suggestive of genomic instability. Consistent with this possibility, the sequence data showed a mis-sense mutation (W701C) in one copy of the IRC20 gene in S/Y_8 (Table S9). IRC20 has a role in homologous recombination and the null mutant is known to have increased Rad52 foci formation (Alvaro *et al.* 2007; Miura *et al.* 2012). Although the IRC20 mutation observed in S/Y_8 is heterozygous, it may contribute to enhanced LOH. Future studies will attempt to test the role of this gene in mitotic genomic instability. It is also possible that enhanced LOH arises from replicative age, wherein daughter cells from older mother cells inherit a hyper-recombination state (McMurray and Gottschling 2003).

We also observed LOH on a whole chromosome-scale-through chromosome loss followed by reduplication (S/Y_7, Figure 2). Such hybrid genome homozygotization for an entire chromosome may happen via mechanisms not explained by classical mitotic recombination pathways. These happen via aberrant chromosome segregation, followed by endoreduplication (Bennett *et al.* 2014). We also observe LOH tracts spanning across the centromere (Figure 2). Since crossovers are suppressed near centromeres in both mitosis and meiosis, our results provide experimental evidence for the occurrence of gene conversions events at centromere locations that are critical for their evolution (Talbert and Henikoff 2010; Thakur and Sanyal 2013; Zafar *et al.* 2017).

Since our experimental strategy involved the use of MA lines, LOH occurs independently of selection and environmental stress that are known to affect LOH rates and types (Forche *et al.* 2011; Bennett *et al.* 2014). Therefore, we can recover LOH events in an unbiased manner to make a LOH map for S288c (Figure 4). A species-wide map shows that all regions across the *S. cerevisiae* pan-genome can undergo LOH (Peter *et al.*, 2018). By analyzing LOH in both S/Y and S/R backgrounds, we made a LOH map specifically for S288c strain (Figure 4). Both known LOH hotspots, as well as new LOH hotspots, were identified from the map. These LOH maps are important as it plays a key role in the development of diseases like cancer (Thiagalingam *et al.* 2002). In conclusion, we show *S. cerevisiae* hybrid genomes are dynamic and highlight the need for analysis of more heterozygous *S. cerevisiae* strains to understand the effects of genetic background on LOH and SNM rates.
